# Mutation of platelet-derived growth factor receptor β causes and exacerbates the severity of brain arteriovenous malformation through enhancing angiogenesis

**DOI:** 10.21203/rs.3.rs-7552935/v1

**Published:** 2025-09-18

**Authors:** Alka Yadav, Leandro Barbosa Do Prado, Mustafa Mohamed, Calvin Wang, Joshua Shi, Zahra Shabani, Rich Liang, Kelly Press, Courtney Tom, Ethan A. Winkler, Hua Su

**Affiliations:** 1 Center for Cerebrovascular Research, Department of Anesthesia and Perioperative Care, University of California, San Francisco, California, USA.; 2 Department of Neurosurgery, University of California, San Francisco, California, USA

**Keywords:** Brain arteriovenous malformation, platelet-derived growth factor receptor-β, pericyte, endoglin, Alk1

## Abstract

Brain arteriovenous malformations (bAVMs) are tangles of abnormal vessels that shunt blood directly from arteries to Veins. The reduction of pericytes is linked to hemorrhage in bAVMs. The PDGFB/PDGFRβ signaling pathway is crucial for regulating pericyte recruitment during angiogenesis. Here, we show that mutation of *Pdgfrβ* causes cerebrovascular malformations in the brain’s angiogenic region, associated with increased pro-angiogenic signaling. Interestingly, the expression of activin receptor-like kinase 1 (Alk1) is decreased in the brain’s angiogenic region of *Pdgfrβ* mutant mice. Overexpression of ALK1 in brain endothelial cells (ECs) reduces angiogenic signaling and the severity of vascular malformations in *Pdgfrβ* mutant mice. Mutation of *Pdgfrβ* also increases bAVM penetrance in endoglin-deficient mice (a gene that causes AVMs), leading to an increase of dysplastic vessels and microhemorrhages in bAVMs. Our data indicate that *Pdgfrβ* mutation causes cerebrovascular malformations and worsens the bAVM phenotype in endoglin mutant mice by enhancing angiogenesis, EC proliferation, and inflammation. Overexpression of ALK1 in brain ECs reduces the severity of cerebrovascular malformations in *Pdgfrβ* mutant mice through downregulating angiogenic signaling.

## Introduction:

An abnormal mass of blood vessels called “nidus” is the main characteristic of brain arteriovenous malformations (bAVMs), which lead to the direct shunting of blood from arteries to veins [1]. Intracranial hemorrhage (ICH) is the most severe complication of bAVMs and the primary reason for treatment. Overall, bAVMs account for 25% of hemorrhagic stroke in children and adults <50 years of age [2]. The cellular and/or molecular mechanisms underlying bAVM destabilization or rupture remained unclear.

Normal cerebrovascular structure and function depend on the coordinated signaling of multiple interconnected cell types, including endothelial cells (ECs), mural cells (vascular smooth muscle cells and pericytes), immune cells, glial cells, and neurons [3,4]. Pericytes are the principal mural cell population of the cerebral microvasculature, covering roughly 80%–90% of the vascular wall [5]. A reduction in the pericytes is associated with both vascular instability and altered hemodynamics in human bAVMs [6,4]. We have observed a mural cell reduction in bAVM with a deficiency of activin receptor-like kinase 1 (*Alk1*, a causative gene for AVM) in mice, which is correlated with the leakage of blood-brain barrier (BBB) [3].

Platelet-derived growth factor-B (PDGFB)/PDGF receptor-β (PDGFR-β) regulates mural cell recruitment during vascular remodeling. We found that Pdgfrβ expression is reduced in *Alk1-deficient* bAVMs [3]. Thalidomide, in a class termed as immunomodulatory drugs (IMIDs), inhibits gastrointestinal bleeding and stabilizes telangiectasia vessels in patients with hereditary hemorrhagic telangiectasis (HHT) through increasing Pdgfb expression [7]. Overexpression of Pdgfb reduced abnormal vessels and hemorrhage in the bAVMs with *Alk1* deletion [8].These data suggest that Pdgfb/Pdgfrβ signaling is impaired in bAVM, leading to a reduction of mural cell coverage of bAVM vessels.

In this study, we tested whether mutation of *Pdgfrβ* alone causes cerebrovascular malformation in mice and increases bAVM severity and the changes of related pathways.

## Material and methods

### Ethics statement

All animal experimental protocols were approved by the Institutional Animal Care and Use Committee (IACUC) of the University of California, San Francisco (UCSF) and conformed to the National Institutes of Health guidelines for the care and use of laboratory animals. Veterinary care was offered by the IACUC faculty. All mice were maintained in a pathogen-free environment and were kept on a 12-hour light-dark cycle with free access to food and water. Animal experiments were conducted by certified investigators who contributed to the study.

### Animals

Five groups of 8- to 10-week-old mice: wild type (WT) mice, *Pdgfrβ* F7^+/−^ and *Pdgfrβ* F7^+/+^ mice that have 7 mutations disrupting Pdgfrβ signaling [9], *Pdgfb*icreER;*Eng*^f/f^ mice that have *Pdgfβ* promoter driving, tamoxifen (TM) inducible cre expression in ECs and a floxed *Eng* gene [10]; and *Pdgfb*icreER;*Eng*^f/f^ ;F7^+/−^ mice were used. A near equal number of male and female mice were included to exclude gender bias.

### Induction of brain angiogenesis and bAVM through stereotactic injection of viral vectors and Intravenous injection of AAV vector

Brain angiogenesis was induced by stereotactic injection of an adeno-associated viral vector (AAV) expressing vascular endothelial growth factor [AAV1-VEGF, 2X10^9^ virus genomes (vgs)] as described in our previous papers [11]. *Pdgfb*icreER;*Eng*^f/f^ and *Pdgfb*icreER;*Eng*^f/f^;F7^+/−^ were treated with TM (2.5 mg/25 g of mouse body weight) for 3 consecutive days starting from the day of AAV1-VEGF injection to delete *Eng* in ECs and to induce bAVM. Brain samples were collected 8 weeks after model induction **(Supplementary Fig 1).**

AAV.cc84-ALK1 (1X10^11^ vgs), a vector that expresses human ALK1 in brain ECs after intravenous injection [11], was delivered via the tail vein a day before the intra-brain injection of AAV1-VEGF. Control mice were injected with AAV.cc84-RFP (1X10^11^ vgs) [11] **(Supplementary Fig 1).**

### Immunofluorescence Staining

Brains were sectioned into 20 μm-thick sections using a Leica CM1950 Cryostat (Leica Microsystems, Wetzlar, Germany). Sections adjacent to the injection site were selected and incubated at 4°C overnight with the following primary antibodies: Rat anti-CD31 antibody (1:100, Cat #SC-18916, Santa Cruz Biotechnology, Santa Cruz, CA) to stain the ECs, Goat anti-CD13 (1:100, Cat # AF2335, R&D Systems, Minneapolis, MN) antibody to stain pericytes, and rabbit anti-mouse α-smooth muscle actin (α-SMA) antibody (1:400, Cat #A2547, Sigma, St Louis, MO) to stain vascular smooth muscle cells. A donkey anti-rat antibody conjugated with Alexa Fluor 488-conjugated (1:100, Cat # A-21208), donkey anti-goat antibody conjugated with Alexa Fluor 594 (1:300, Cat #A-11058), donkey anti-rabbit antibody conjugated with Alexa Fluor 555 (1:400, Cat #A-31572, Thermo Fisher Scientific, Waltham, MA), were used as the secondary antibodies to visualize positive stains.

Sections were mounted with Vectashield antifade DAPI containing mounting medium (Cat #H-1200, Vector Laboratories, Burlingame, CA). Images were taken using a Keyence microscope (Keyence, Model BZ-9000, Itasca, IL). Vascular density and vascular pericyte coverage were quantified using NIH Image 1.63 software. Dysplastic vessels and SMA-positive vessels were counted manually. Dysplasia index (the number of vessels with a lumen diameter larger than 15 μm/mm^2^) was used to quantify abnormal vessels.

### Latex perfusion

Blue latex dye (Catalog BR80B, Connecticut Valley Biological Supply Co., Southampton, MA, USA) perfusion was done as described previously [12].

### Prussian blue staining

Two sections per brain, adjacent to the injection site were used to detect iron deposition using an Iron Stain Kit (Sigma-Aldrich, St. Louis, MO) according to the manufacturer’s instructions. The positively stained areas (blue) on the sections were quantified using NIH Image 1.63 software.

### RNA isolation, RNA sequencing (RNAseq), and quantitative reverse transcriptase polymerase chain reaction (qRT-PCR).

Total RNA was isolated and purified from 2 mm^3^ tissues around the AAV1-VEGF injected sites using TRIzol (Invitrogen, Carlsbad, CA), followed by RNeasy (QIAGEN, Germantown, MD) binding and quantified by a NanoDrop^™^ Lite (ThermoFisher Scientific, Waltham, MA, USA). A 0.2 μg RNA from each sample was sent to Novogene Co. (Sacramento, CA) for RNAseq using the company’s standard protocol **(Supplemental file S1).** Novogene Co analyzed the outcome data.

Differential expression analysis was used to compare the differences in gene expression across the experimental groups and to detect gene expression changes predominantly seen in specific experimental groups. Gene Ontology (GO) and Kyoto Encyclopedia of Genes and Genomes (KEGG) analyses were used to detect the molecular and signaling pathway changes among groups.

For qRT-PCR, complementary DNA (cDNA) was prepared from 2 μg total RNA using the SuperScript III First-Strand Synthesis System (Invitrogen, Carlsbad, CA). The TaqMan primers/probes, Vegfa (Catalog Mm01281449), Alk1 (Catalog Mm00437432_m1), Kdr (Mm01222421), and Akt3 (Mm01311133_m1, Thermo Fisher Scientific) were used to analyze gene expression. PCR was performed using cDNA 10 ng, 50 nmol of each primer, and Taqman Fast advanced Master Mix (Catalog number 4444963, Thermo Fisher Scientific) in 20 μl reactions following the manufacturer’s protocol on an Agilent Mx3005P Real-Time PCR system (Agilent Technologies, Santa Clara, CA).Data were normalized using endogenous control HPRT (Mm01545399_m1) or GAPDH (Catalog Mm02758991_g1, Thermo Fisher Scientific).

### Statistics

Sample sizes were calculated based on prior data with the following assumptions: α = 0.05, β = 0.2 (power 80%). For quantification, sections were randomized and analyzed by two researchers blinded to the experimental groups. Inter-observer discrepancy was controlled within one standard deviation. Data are presented as mean ± standard deviation (SD). Non-normally distributed data were log-transformed before analysis. All data were analyzed using a t-test for two-sample comparison or one-way ANOVA for multiple-sample comparison, followed by Tukey’s multiple comparisons using GraphPad Prism 10 software. A *p*-value < 0.05 was significant. Sample sizes are indicated in figure legends.

## Results

### 1. *Pdgfrβ* F7 mutation induced pericyte loss and cerebrovascular malformation in mice

**Fig 1.**
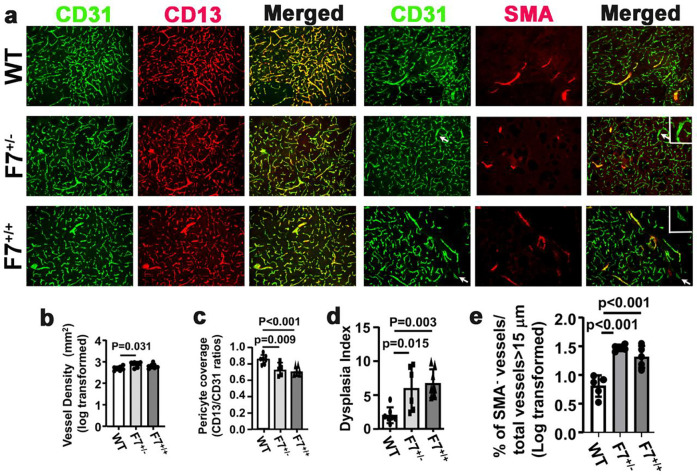
*Pdgfrβ* F7 mutation induced pericyte loss and cerebrovascular malformation in mice. **a.** Representative images of brain sections. ECs were stained green. Pericytes were stained red. Vascular smooth muscles were stained red. Arrows indicate abnormal vessels. Scale bar = 50 μm. Inserts show enlarged abnormal vessels indicated by arrows in the pictures that lack smooth muscle coverage. b-e are the quantification of **v**ascular density (**b**), pericyte coverage (**c**), dysplasia index (**d**), and % of SMA^−^ vessels/total vessels > 15 μm (**e**). n=6–7.

**Fig 2.**
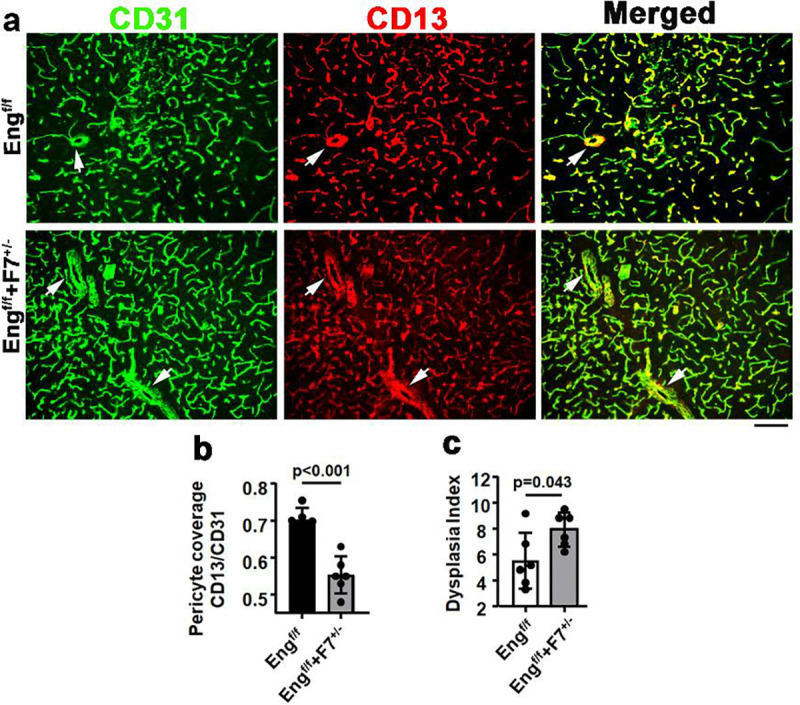
*Pdgfrβ* F7 mutation increases dysplastic vessels in the bAVMs of *Eng* mutant mice. **a.** Representative images of brain sections. ECs were stained green. Pericytes were stained red. Arrows indicate abnormal vessels. Scale bar = 50 μm. **b** and **c** are quantifications of Pericyte coverage **(b)** and dysplasia index **(c)**. Eng^f/f^: *Pdgfb*icreER;*Eng*^f/f^ mice; *Eng*^f/f^+F7^+/−^: *Pdgfbicre*ER;*Eng*^f/f^,F7^+/−^ mice. n=6

To test if mutation of *Pdgfrβ* alone causes bAVM formation, we induced brain focal angiogenesis in WT, *Pdgfrβ* F7^*+/−*^ (F7^*+/−*^) and *Pdgfrβ* F7^*+/+*^ (F7^*+/+*^) mice and analyzed vascular mural cell-coverage, vessel density, and dysplasia index on brain sections co-stained with CD31 and CD13 antibodies, and CD31 and α-SMA antibodies (Fig 1a). Compared to WT mice, F7^+/−^ mice had more vessel density **(p=0.030,** Fig 1b), low pericyte coverage in F7^+/−^ **(p=0.009)** and F7^+/+^ **(p<0.001,** Fig 1c). The pericyte coverages in F7^+/+^ and F7^+/−^ mice were similar (**p=0.54,** Fig. 2c). F7^+/−^ **(p=0.015)** and F7^+/+^ **(p=0.003)** had more dysplastic vessels than WT mice (Fig. 2d) and more vascular smooth muscle negative vessels than WT mice **(p<0.001,** Fig 1e). Vascular densities were similar between the two groups. *Pdgfrβ* F7 mutation also leads to hemorrhage in the brain angiogenic region (**Suppl Fig 2**). We have observed more AVM-like vessels in F7 mice **(Suppl Fig 3)**.

### 2. *Pdgfrβ* F7 mutation reduced vascular pericyte coverage, increased dysplastic vessels in bAVMs of *Eng* EC deleted mice

To test whether the mutation of *Pdgfrβ* enhances the severity in Eng EC deleted mice. We induced bAVMs in *Pdgfb*icreER;*Eng*^f/f^ and *Pdgfb*icreER;*Eng*^f/f^;F7^+/−^ mice and analyzed vascular mural cell-coverage and quantified the dysplasia index on brain sections stained with CD31 and CD13 antibodies (Fig 2a) and CD31 and α-SMA antibodies. We found that compared to *Pdgfbicre*ER;Eng^f/f^ mice, *Pdgfbicre*ER;Eng^f/f^,F7^+/−^ mice had less vascular pericyte coverage **(p<0.001,** Fig 2b), more dysplastic vessels **(p=0.043,** Fig 2c). Mutation of *Pdgfrβ* did not alter the vessel density (**p=0.598**) and smooth muscle coverage (**p=0.055**) in *Eng*-deficient bAVM. Microhemorrhages were detected in the bAVM lesions of *Pdgfbicre*ER;*Eng*^f/f^ mice and *Pdgfbicre*ER;*Eng*^f/f^,F7^+/−^ mice (**Suppl Fig 2**). *Pdgfrb* F7 mutation did not increase hemorrhage in the bAVM of *Pdgfbicre*ER;Eng^f/f^ mice (**Suppl Fig 2**). We have observed more AVM-like vessels in latex perfused *Pdgfbicre*ER;*Eng*^f/f^,F7^+/−^ mice than *Pdgfbicre*ER;*Eng*^f/f^ mice **(Suppl Fig 3)**.

### 3. *Pdgfrβ* F7 mutation increased the pro-angiogenic and EC proliferation signaling and inflammation in the brain angiogenic regions and bAVMs of *Eng* mutant mice

**Fig 3.**
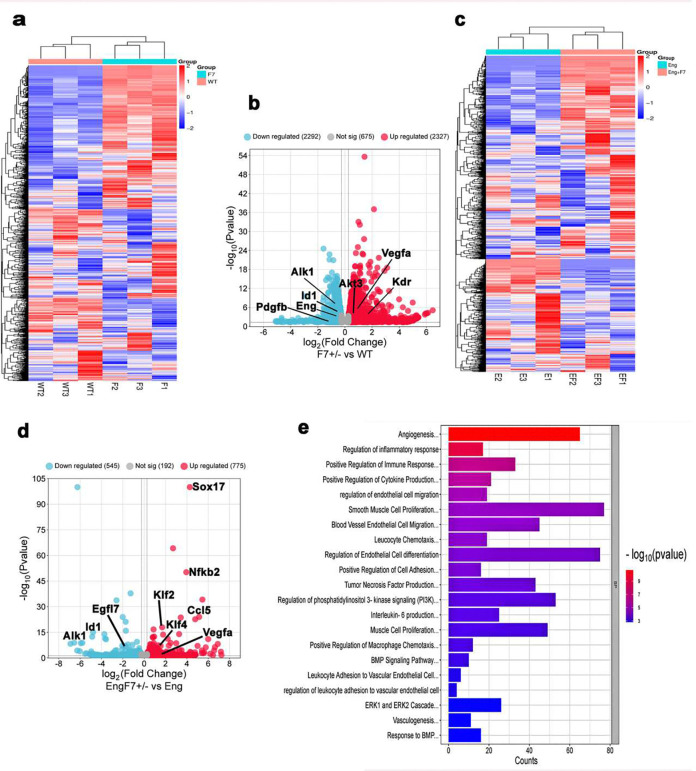
*Pdgfrβ* F7 mutation increased the expression of pro-angiogenic and pro-inflammatory gene expression in the brain angiogenic region and bAVM of *Eng mutant* mice. **(a)** Heatmap showing differential expression of genes in the brain angiogenic regions of WT mice (WT1, WT2, WT3) and *Pdgfrβ* F7^+/−^ mutant mice (F1, F2, F3). **(b)** Volcano plots showing upregulated (red) and downregulated (blue) genes in the brain angiogenic regions of *Pdgfrβ* F7^+/−^ mutant mice (F7) compared to WT mice. **(c)** Heatmap showing differential expression of genes in the bAVMs of *Pdgrb*icreER;*Eng*^f/f^ mice (E1, E2, E3) and *Pdgrb*icreER;*Eng*^f/f^;F7^+−^ mice (EF1, EF2, and EF3). **(d)** Volcano plots showing upregulated (red) and downregulated (blue) genes induced by *Pdgfrβ* F7^+/−^ mutant in the bAVMs of *Pdgrb*icreER;*Eng*^f/f^ mice. ENG+F7: *Pdgrb*icreER;*Eng*^f/f^+F7^+/−^ mice; ENG: *Pdgrb*icreER;*Eng*^f/f^ mice. (**e)** Top changed biological pathways induced by *Pdgfrβ* F7^+/−^ mutant in the bAVMs of *Pdgrb*icreER;*Eng*^f/f^ mice.

To investigate the influence of *Pdgfrβ* F7 mutation in RNA transcriptomics in the brain angiogenic region and bAVM of *Pdgfb*icreER;*Eng*^f/f^ mice, we performed RNA-seq. Differential analysis showed a distinctive gene expression profile in the brain angiogenic region of WT mice and *pdgfrβ* F7 mutation mice (Fig 3a). *Pdgfrβ* F7 mutation upregulated the expression of pro-angiogenic genes, Vegfa, Kdr, and Akt3, and downregulated genes related to pericyte recruitment and *Tgfβ1* signaling, *Pdgfb*, *Alk1*, *Eng*, and *Id1* (Fig 3b, **Suppl Fig 4 & Suppl Table 1**). *Pdgfrβ* F7 mutation upregulated phosphatidylinositol metabolic process;PI3K (KEGG analysis, adj. p=0.008) and TOR signaling (GO analysis, Adj. p=0.020).

*Pdgfrβ* F7 mutation also induced distinctive changes in gene expression (Fig 3c) and transcriptional changes in the bAVM of *Pdgfb*icreER;*Eng*^f/f^ mice, increased angiogenic and proinflammatory gene expression and signaling (Fig 3d & e, **Suppl Table 2 and 3**).

Together, these findings suggest that the *Pdgfrβ* F7 mutation enhances angiogenic and inflammatory signaling pathways in the brain angiogenic region and *Eng*-deficient bAVMs, leading to a more severe bAVM phenotype.

### 4. Overexpression of ALK1 in brain ECs reduced the severity of cerebrovascular malformations in *Pdgfrβ* F7 mutant mice.

**Fig 4.**
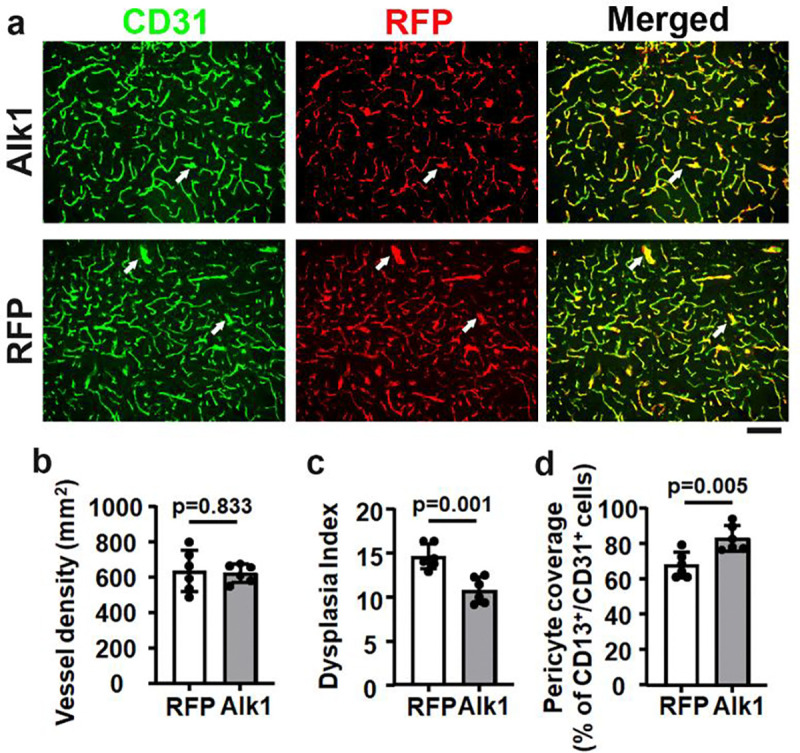
Overexpression of ALK1 in the brain ECs reduced the severity of cerebrovascular malformation in *Pdgfrβ* F7 mutant mice. **a.** Representative images of brain sections. ECs were stained green. Pericytes were stained red. Arrows indicate abnormal vessels. Scale bar = 50 μm. **b-d** are the quantification of **v**ascular density (**b**), dysplasia index (**c**), and pericyte coverage (**d**). n=6.

Interestingly, *Pdgfrβ* F7 mutation reduced Alk1 expression in the brain angiogenic region. Since the mutation of the *Alk1* gene in ECs leads to AVM development [13], we tested whether overexpression of ALK1 (human gene) in brain ECs reduces the severity of cerebrovascular malformation in *Pdgfrβ* F7 mutant mice. We used an AAV vector, AAV.cc84, which we have developed, that can transduce brain ECs through intravenous injection [11] to deliver the ALK1 gene into brain ECs of *Pdgfrβ* F7 mutant mice. We found that overexpression of ALK1 in brain ECs reduced the dysplasia index and increased pericyte coverage in the brain angiogenic region of *Pdgfrβ* F7 mutant mice (Fig 4). Overexpression of ALK1 in brain ECs has also reduced pro-angiogenic gene expression (**Suppl Fig. 4**).

These data indicated that overexpression of ALK1 in brain endothelial cells of *Pdgfrβ* F7 mutant mice reduces the severity of cerebrovascular malformation through downregulation of angiogenesis.

## Discussion

In this study, we found that inactivation of the main downstream signaling pathways of PDGFRβ via the F7 mutation triggers cerebrovascular malformations following angiogenic stimulation. *Pdgfrβ* F7 mutation also enhanced the severity of bAVM in *Eng* mutant mice through upregulating pro-angiogenic and pro-inflammatory genes and pathways. *Pdgfrβ* F7 mutation reduced the expression of several genes involved in Tgfβ1 signaling, including *Alk1* and *Eng*, both are AVM causative genes, in the brain angiogenic region. Overexpression of the human ALK1 gene in brain ECs reduces the severity of cerebrovascular malformation, suggesting that downregulation of Tgfβ1 signaling is one of the mechanisms underlying the cerebrovascular malformations induced by *Pdgfrβ* F7 mutation.

PDGFRβ is expressed in multiple cell types, including pericytes, vSMCs, and neurons [14]. Its ligand, PDGFB, is secreted from the ECs of angiogenic sprouts, where it works as an attractant for pericytes. Homozygous deletion of *Pdgfb* or *Pdgfrβ* in rodents results in high embryonic mortality due to widespread hemorrhage[15]. Disruption of PDGFB/PDGFRβ signaling also causes excessive vascular abnormalities and microaneurysms [16]. *Pdgfrβ* F7 mutant mice have less pericyte coverage in the brain and spinal cord of adult 6–8-month-old mice [5]. Consistent with these findings, we showed a significant reduction of pericyte coverage, abnormal vessel morphology, and microhemorrhages in the brain angiogenic region of *Pdgfrβ* F7 mutant mice. In addition, we showed that Pdgfrβ F7 mutations upregulate the expression of pro-angiogenic genes and downregulate the expression of genes related to Tgfβ1 signaling in the brain angiogenic region.

Abnormal vascular remodeling and vascular instability are associated with bAVM development and progression [4]. Abnormal expression of PDGFB and PDGFRβ has been detected in bAVMs in humans and rodents [6,3]. Pdgfrβ expression was reduced in the bAVM lesions of *Alk1* mutant mice, which was associated with a reduction of mural cell coverage [3]. We showed that Alk1 expression is reduced in the brain angiogenic region of *Pdgfrβ* F7 mutant mice, which is associated with the development of cerebrovascular malformations. Overexpression of human ALK1 in brain ECs reduced the severity of vascular malformation in the brain angiogenic region of *Pdgfrβ* F7 mutant mice. These findings suggest a possible crosstalk between ALK1 and PDGFRβ. However, direct binding between these two receptors has not been identified. Currently, it is understood that ALK1 activity in ECs regulates PDGFB production, which subsequently signals through PDGFRβ in pericytes. The mechanism by which PDGFRβ affects ALK1 expression or function remains unclear and needs to be studied further.

Reduction of mural cell coverage in the bAVM vessels in mouse models and human specimens is associated with the reduction of PDGFB and PDGFRβ protein levels in bAVM lesions and bAVM bleeding [6,3]. The role of PDGFB/PDGFRβ signaling in human bAVM pathogenesis has not been studied fully. In this study, we tested the influence of *Pdgfrβ* F7 mutant on bAVMs using a mouse bAVM model we have developed by deletion of *Eng* in ECs plus focal angiogenic stimulation [12]. We found that *Pdgfrβ* F7 mutant increases the number of abnormal vessels and reduces vascular pericyte coverage in the bAVMs of *Eng*-deficient mice, which is associated with increased pro-angiogenic and pro-inflammatory signaling. These data indicate that Pdgfrβ plays roles in bAVM pathogenesis.

Interestingly, *Pdgfrβ* F7 mutation decreased the expression of Alk1 and Eng in the brain’s angiogenic region, and Alk1 expression in the bAVMs of Eng-deficient mice, indicating a regulatory role of Pdgfrβ on Alk1 and Eng expression. To test whether reducing Alk1 expression contributes to cerebrovascular malformation in *Pdgfrβ* F7 mutant mice, we overexpressed a human ALK1 gene in brain ECs of *Pdgfrβ* F7 mutant mice. We found that overexpression of ALK1 in brain ECs reduces the expression of pro-angiogenic genes and the severity of cerebrovascular malformation in the brain’s angiogenic region in *Pdgfrβ* F7 mutant mice.

During angiogenesis, pericyte recruitment to EC tubes is orchestrated by several signaling cascades, including PDGFB/PDGFRβ, TGFβ, Notch, VEGF, and angiopoietin pathways [17]. Disruption of any component of this signaling network can impair pericyte recruitment and vascular stability. We showed in addition to reducing mural cell recruitment, the *Pdgfrβ* F7 mutation induced expression of genes associated with angiogenesis, EC proliferation, and inflammation in *Eng*-deficient bAVMs. Co-mutation of *Pdgfrβ* and *Eng* not only disrupts mural cell recruitment but also creates a pro-angiogenic and pro-inflammatory microenvironment that drives bAVM progression. Furthermore, chronic inflammation in this context likely acts in a feedforward loop, where increased cytokines and chemokines further reduce pericyte recruitment and exacerbate vascular instability. Taken together, our findings establish a mechanistic link between PDGFRβ mutation and the development of vascular malformations, highlighting the critical interplay between pericyte recruitment, angiogenic signaling, and inflammatory processes in bAVM pathogenesis.

Notably, overexpression of ALK1 in brain ECs ameliorates the vascular abnormalities induced by *Pdgfrβ* F7 mutation. Thus, ALK1 is not only a downstream effector influenced by PDGFRβ activity but also a valuable therapeutic target for restoring vascular stability. Pharmacological agents or gene therapy approaches aimed at enhancing ALK1 expression or signaling may offer a promising strategy to mitigate bAVM severity, especially in individuals with dysfunctions of PDGFB/PDGFRβ and TFGβ pathways. Future studies should focus on elucidating the molecular crosstalk between PDGFRβ and ALK1, and on developing targeted therapies that restore balance to this signaling axis in cerebrovascular disease.

## Conclusion:

This study shows that the *Pdgfrβ* F7 mutation alone induces vascular malformations by increasing pro-angiogenic gene expression and suppressing TGFβ1 signaling. *Pdgfrβ* F7 mutation also worsens the severity of bAVMs in *Eng*-deficient mice via enhancing angiogenic and inflammatory pathways. ALK1 overexpression in brain ECs mitigates these defects, suggesting its therapeutic potential. Future research should investigate cell-specific roles of PDGFRβ and strategies to restore pericyte recruitment.

## Supplementary Material

This is a list of supplementary files associated with this preprint. Click to download.

• SupplementaryFile5ForSubmission.docx
